# Biomarkers of collagen turnover are related to annual change in FEV_1_ in patients with chronic obstructive pulmonary disease within the ECLIPSE study

**DOI:** 10.1186/s12890-017-0505-4

**Published:** 2017-12-04

**Authors:** Diana J. Leeming, Inger Byrjalsen, Jannie M. B. Sand, Asger R. Bihlet, Peter Lange, The Evaluation of COPD Longitudinally to Identify Surrogate Endpoints (ECLIPSE) study investigators, H. Coxson, L. Edwards, R. Tal-Singer, D. Lomas, W. MacNee, E. Silverman, C. Crim, J. Vestbo, J. Yates, A. Agusti, P. Calverley, B. Celli, C. Crim, B. Miller, W. MacNee, S. Rennard, R. Tal-Singer, E. Wouters, J. Yates, Y. Ivanov, K. Kostov, J. Bourbeau, M. Fitzgerald, P. Hernandez, K. Killian, R. Levy, F. Maltais, D. O’Donnell, J. Krepelka, J. Vestbo, E. Wouters, D. Quinn, P. Bakke, M. Kosnik, A. Agusti, J. Sauleda, P. de Mallorca, Y. Feschenko, V. Gavrisyuk, L. Yashina, N. Monogarova, P. Calverley, D. Lomas, W. MacNee, D. Singh, J. Wedzicha, A. Anzueto, S. Braman, R. Casaburi, B. Celli, G. Giessel, M. Gotfried, G. Greenwald, N. Hanania, D. Mahler, B. Make, S. Rennard, C. Rochester, P. Scanlon, D. Schuller, F. Sciurba, A. Sharafkhaneh, T. Siler, E. Silverman, A. Wanner, R. Wise, R. ZuWallack, Ruth Thal-Singer, Bruce E. Miller, Morten A. Karsdal, Jørgen Vestbo

**Affiliations:** 1 Nordic Bioscience, Fibrosis Biology and Biomarkers, Herlev Hovedgade 207, DK-2730 Herlev, Denmark; 2 Section of Social Medicine, Institute of Public Health, University of Copenhagen, Copenhagen, Denmark; 3 GlaxoSmithKline Research and Development, King of Prussia, PA United States; 4 Centre for Respiratory Medicine and Allergy, Manchester Academic Science Centre, The University of Manchester and University Hospital South Manchester NHS Foundation Trust, Manchester, UK

**Keywords:** COPD, Lung function change, Prognosis, Serological marker, Extracellular matrix

## Abstract

**Background:**

Change in forced expiratory volume in one second (FEV_1_) is important for defining severity of chronic obstructive pulmonary disease (COPD). Serological neoepitope markers of collagen turnover may predict rate of change in FEV_1_.

**Methods:**

One thousand COPD subjects from the observational, multicentre, three-year ECLIPSE (Evaluation of COPD Longitudinally to Identify Predictive Surrogate Endpoints) study (NCT00292552, trial registration in February 2006) were included. Matrix metalloproteinase (MMP)-generated fragments of collagen type I, and type VI (C1M and C6M) were assessed in month six serum samples. A random-coefficient model with both a random intercept and a random slope was used to test the ability of the markers to predict post-dose bronchodilator FEV_1_ (PD-FEV_1_) change over two years adjusting for sex, age, BMI, smoking, bronchodilator reversibility, prior exacerbations, emphysema and chronic bronchitis status at baseline.

**Results:**

Annual change of PD-FEV_1_ was estimated from a linear model for the two-year study period. Serum C1M and C6M were independent predictors of lung function change (*p* = 0.007/0.005). Smoking, bronchodilator reversibility, plasma hsCRP and emphysema were also significant predictors. The effect estimate between annual change in PD-FEV_1_ per one standard deviation (1SD) increase of C1M and C6M was +10.4 mL/yr. and +8.6 mL/yr. C1M, and C6M, had a significant association with baseline FEV_1_.

**Conclusion:**

We demonstrated that markers of tissue turnover were significantly associated with lung function change. These markers may function as prognostic biomarkers and possibly as efficacy biomarkers in clinical trials focusing on lung function change in COPD.

**Trial registration:**

NCT00292552, Retrospectively registered, trial registration in February 2006.

**Electronic supplementary material:**

The online version of this article (10.1186/s12890-017-0505-4) contains supplementary material, which is available to authorized users.

## Background

Chronic obstructive pulmonary disease (COPD) is a leading cause of morbidity and mortality worldwide and is a disease leading to narrowing of the small airways, airway fibrosis and chronic changes in the lung parenchyma [1] mainly due to cigarette- or biomass smoke exposure [2]. Phenotyping of patients is generally based on a combination of clinical and morphological features such as frequency of exacerbations, lung function, presence or absence of emphysema and chronic bronchitis, and presence of asthmatic features [3, 4]. Therapies affecting such parameters in COPD are limited [5].

In COPD, the airway composition and structure is changed compared to the healthy state, due to small airway inflammation resulting in epithelial damage and subsequent airway fibrosis with increased deposition and remodelling of extracellular matrix (ECM) proteins in the basement membrane and interstitial matrix [6–8] of the lung. Activated lung fibroblasts deposit ECM proteins, such as collagens type I and III, fibronectin, laminin [7, 9–11], biglycan, decorin and perlecan [12], all alleged of having severe effects on the lung integrity. These proteins are believed to be elevated and remodelled during chronic inflammation in the small airways. Furthermore, pathological proteases such as elastase [13], and matrix metalloproteinase (MMP)-1, −2, −7, −9 and −12 [14] have been reported to be over-expressed in COPD affected tissue [15]. The most abundant collagen in the interstitial matrix of the lung is the fibril-forming type I collagen [16] which is mainly found located alongside with type III collagen, the second most abundant collagen type. Together they provide the structural framework of the alveolar wall, pulmonary blood vessels, visceral pleura and the connective tissue sheaths that surround the tracheobronchial tree [17, 18]. Type VI collagen is another important member of the collagens of the interstitial matrix, which main function is to connect cells to territorial matrix [19, 20]. Recently, it was shown that circulating neoepitope markers of ECM were related to clinical outcomes in patients with idiopathic pulmonary fibrosis (IPF) [21]. In IPF, serum markers of MMP-generated fragments of type I, III, VI collagen, biglycan and CRP were able to identify patients with fast progression and high risk of mortality. Generally, markers of ECM remodellings including C1M and C6M have been shown to relate to disease activity in the osteoporosis, COPD and pagets disease, rheumatoid arthritis (RA), hepatitis C, hepatitis B [22–31] C6M and C1M has been shown to be elevated in COPD compared to healthy individuals; C6M is upregulated during AECOPD, however not related to smoking status or pack years [32–35]. In the present work, we aimed to investigate the ability of novel serological ECM related neoepitope markers to predict rate of change of forced expiratory volume in one second (FEV_1_) in COPD patients within the ECLIPSE study [36]. The markers utilized are highly specific for the detection of MMP-mediated fragments of type I (C1M [37]) or type VI collagen (C6M [19]). The hypothesis was that some of these ECM markers, which are markers of pulmonary fibrosis in patient with idiopathic pulmonary fibrosis (IPF) [21], were able to predict lung function change in patients with pulmonary structural changes due to COPD. We speculated that these markers may be valid as an aid in the selection of patients in most need of treatment, and patients for clinical trials focusing on progression of COPD.

## Methods

### Study design

The present analysis was performed in the three-year ECLIPSE observational study (ClinicalTrials.gov number, NCT00292552, trial registration in February 2006) described previously [36, 38]. The full ECLIPSE study included 2164 patients with COPD. Briefly, patients included were between 40 and 75 years at baseline, had a history of ten or more pack-years of smoking, a post-dose bronchodilator FEV_1_ less than 80% of the predicted value and a post-dose bronchodilator ratio of FEV_1_ to forced vital capacity (FVC) of 0.7 or less. The Global initiative for Obstructive Lung Disease (GOLD) spirometry stages included were II-IV. Respiratory symptoms, smoking history, occupational exposure, and coexisting medical conditions were documented at study entry with the use of a modified version of the American Thoracic Society–Division of Lung Disease (ATS-DLD) questionnaire. The study was conducted in accordance with the Declaration of Helsinki and good clinical practice guidelines, and was approved by all local and participating centres’ ethics and review boards reference number SCO104960 (ECLIPSE) RM 2005/00273/05 (Additional file 1). All patients provided their informed written consent**.** A list of the particpating centers is seen in Additional file 1. Each patient underwent spirometry before and 15 min after inhaling 400 μg of salbutamol (GlaxoSmithKline). Computed tomographic (CT) scanning of the chest was performed at baseline to evaluate the severity and distribution of emphysema. Quantitative assessment of lung volumes and estimation of the percentage of lung CT voxels below a threshold of −950 Hounsfield units and percent low areas of attenuation (%LAA) was performed using Pulmonary Workstation software, version 2.0 (VIDA Diagnostics). Significant amount of emphysema was defined as having more than 10%LAA. Whole blood was collected by venipuncture into vacutainer tubes. Serum was prepared by allowing the blood to clot for 30 min at room temperature followed by centrifugation at 1500 g for 10–15 min. Plasma was obtained by centrifugation of vacutainer tubes at 2000 g for 10–15 min.

### Definition of subpopulation

**Fig. 1:**
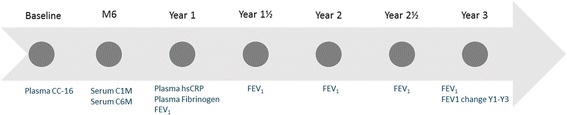
Histogram showing the distribution of annual FEV_1_ change in the presented subpopulation of ECLIPSE

A subpopulation of 1000 patients was selected from the ECLIPSE cohort for the purpose of evaluating the ability of two serum markers of ECM remodeling to predict the longitudinal change in FEV_1_ within the study compared to existing biomarkers assessed within the study. The 500 most rapid post-dose bronchodilator FEV_1_ (PD-FEV_1_) decliners and the 500 slowest PD-FEV_1_ decliners were selected for the biomarker assessments. The linear PD-FEV_1_ decline was defined as the change in PD-FEV_1_ from year 1 (Y1) to year 3 (Y3). Baseline to month 6 (M6) PD-FEV_1_ data were excluded in the present analysis since the biochemical markers included in the present study analysis were assessed in serological samples at different time points: baseline (BL), M6 or Y1. A schematic overview of the data used is seen in Fig. 1. This way only PD-FEV_1_ data obtained post blood sampling were used in the analysis.

### Quantification of biochemical markers in the subpopulation

Serum samples from the M6 visit that had been stored at −80 °C until used for analysis. Serum levels of a MMP derived collagen type I fragment (C1M) [37] and a MMP derived collagen type VI fragment (C6M) [19] were assessed using competitive enzyme linked immunosorbent assays (ELISAs) (Nordic Bioscience, Herlev, DK) in the subpopulation of 1000 patients. Technical data on robustness, specificity and analyte stability are published for C1M and C6M [37, 19]. Biomarkers were assessed in a blinded manner according to the manufacturer and all samples were measured within the detection range. EDTA plasma levels of CC-16 at BL, hsCRP and fibrinogen at Y1 had prior to this study been assessed in the total ECLIPSE cohort [39]. Thus data of CC-16, hsCRP and fibrinogen from the ECLIPSE database were used for analysis.

### Statistical analysis

Demographic characteristics have been summarized as mean with standard deviation (SD) or number of subjects with percentage as applicable. The change in FEV_1_ over time was estimated from five FEV_1_ measurements performed through Y1 to Y3 in the study using a linear regression model. Negative PD-FEV_1_ refers to a loss of FEV_1_ and positive PD-FEV_1_ refers to a retaining of PD-FEV_1_. A saddle test was performed (proc modeclus) on all FEV_1_ change data using a radius of the sphere of support for uniform-kernel density estimation and the neighborhood for clustering of *R* = 10 or *R* = 20. The association of PD-FEV_1_ and PD-FEV_1_ in percent of predicted value (PD-FEV_1_%) was calculated using a random-coefficient model with both a random intercept and a random slope including the co-variates gender, age, BMI, current smoking status, bronchodilator reversibility, number of prior acute exacerbations, emphysema, chronic bronchitis status, and number of pack-years at baseline. A multimarker random-coefficient model including C1M, C6M, fibrinogen, and hsCRP in one model or using stepwise exclusion tested for association to the linear change of PD-FEV1 was performed. Furthermore, the association to the linear change of PD-FEV1 was calculated for the total population; in patients stratified according to GOLD stages B, C and D; and in patients defined as slow or rapid decliners using a cut-off value of FEV_1_ of 40 mL/yr. Levels of C1M, C6M and hsCRP in patients with or without significant emphysema (%LAA < 10) were tested using a Mann-Whitney unpaired t-test. The SAS software package (release 9.3; SAS Institute Inc., Cary, NC, USA) was used for the statistical calculations.

### Funding source

The study was sponsored by GlaxoSmithKline; the Danish Agency for Science, Technology and Innovation; and the Danish Research Foundation. Two representatives of GlaxoSmithKline and one academic, together representing the ECLIPSE study investigators, two representatives of Nordic Bioscience developed the present study design and concept. All approved the plan for the current analyses, had full access to the data, and were responsible for the decision to publish. The study sponsor did not place any restrictions with regard to statements made in the final paper.

## Results

**Fig. 2:**
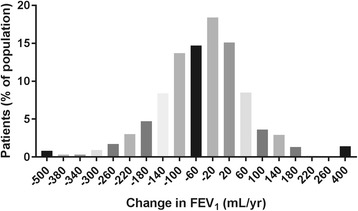
Schematic overview of data used from the ECLIPSE study for analysis in the present study. M6 = Month 6 time point

The demographic description of the patient population is presented in Table 1. The mean annual rates of change in PD-FEV_1_ and PD-FEV_1_% in all patients from year 1 to year 3 were −43 ± 120 mL and −1.5 ± 4.2%, respectively. The mean annual rates of change were −132 ± 91 mL and −4.7 ± 3% for PD-FEV_1_ and PD-FEV_1_% in the 500 most rapid decliners and +44 ± 95 mL and +1.6 ± 3% in the slowest decliners. The distribution of estimated FEV_1_ change (Fig. 2) showed that most patients lost between −20 and −200 mL/yr. (60%) or gained 20–160 mL/yr. (30%) in FEV_1_ in the two-year period. A saddle test of the estimated FEV_1_ change distribution did not indicate that this parameter followed a bi-phasic distribution. Patients in the population did genereally have a stable annual rate of change in FEV_1_. C1M, C6M and hsCRP were not significantly elevated in patients with significant emphysema (%LAA > 10) compared to those without.

**Table 1:** Population demographics at baseline

Variable	N	Mean	SD	SEM
Age (yr)	1000	63.1	7.21	0.2
BMI	1000	26.9	5.9	0.2
Gender (F/M)	363/637	–	–	–
GOLD stage	II(494), III(406), IV(100)	–	–	–
PD-FEV_1_%	998	50.6	15.2	0.5
%LAA	887	16.3	11.3	0.4

*F* Female, *M* Male, *PD-FEV*_*1*_ *%* Percent predicted normal post dose forced expiratory volume in 1 s, *%LAA* Percent low areas of attenuation below −950 Hounsfield units, *BMI* Body mass index

### Association between clinical patient characteristics or single biomarkers and baseline FEV_1_

**Fig. 3:**
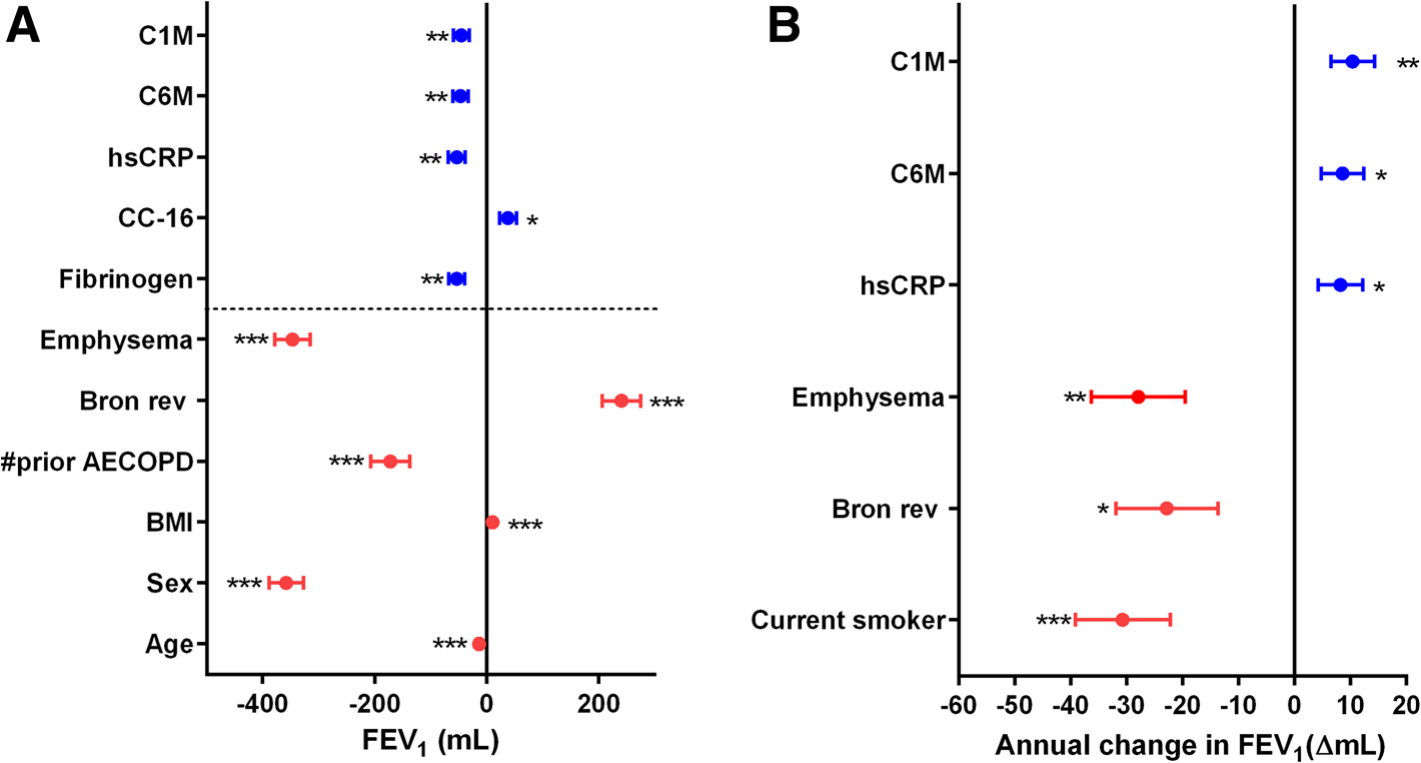
Forest plot of associated effects of serological markers (*blue*) or patient characteristics (*red*) to describe (**a**) baseline FEV_1_ or (**b**) predict annual change in FEV_1_. The number of mL or change in mL/yr. are based on one standard deviation change for serological biomarkers, yes versus no for emphysema, bronchodilator reversibility (bron rev), emphysema, current smoker; per year for age; Female versus male for sex; or kg/m^2^ for BMI. Number of prior exacerbations (#prior AECOPD) shown here is one versus zero. Data are shown as mean ± standard error range

There was a statistical significant association between a range of patient characteristics or biomarkers and baseline PD-FEV_1_ (Table 2). Age, BMI, sex, emphysema, bronchodilator reversibility and no prior exacerbation showed the most pronounced association (*p* < 0.0001) in the total population. An overview of the association for significant parameters is seen in Fig. 3, showing that both patient characteristics and biomarkers were able to describe baseline FEV_1_ difference. The association of a 1SD change in a biomarker was calculated for each marker in the subpopulation for PD-FEV_1_ showing associations between −53.3 to 38.1 mL (Table 2). Plasma fibrinogen and hsCRP had the largest association (−53.3/−53.3 mL, *p* = 0.0002/0.0003), and serum C1M and C6M also showed significant association (−45.2 mL, *p* = 0.002; −46.5 mL, *p* = 0.0011) to baseline FEV_1_.

**Table 2:** Association between patients’ characteristics and biomarkers on baseline Forced Expiratory Volume in 1 Second (FEV_1_) assessed in millilitres (mL) and annual rate of change in FEV_1_. Data are shown as mean ± standard error (SE). Data are generated from a random-coefficient model. The *p*-values are not corrected for multiple testing

Marker	Association^a^ with baseline FEV_1_	*P* value	Association^a^ with annual rate of change in FEV_1_	*P* value
	mL		mL/yr	
Serological biomarkers				
BL CC-16	38.1 ± 15.2	***0.012***	−3.0 ± 4.0	0.50
BL SP-D	7.6 ± 14.4	0.60	−2.9 ± 3.8	0.50
BL WBC	−24.0 ± 14	0.09	−2.8 ± 3.8	0.50
MO_6_ C1M	−45.2 ± 14.4	***0.002***	10.4 ± 3.9	***0.007***
MO_6_ C6M	−46.5 ± 14.2	***0.0011***	8.6 ± 3.8	***0.03***
Y1 hsCRP	−53.3 ± 14.7	***0.0003***	8.2 ± 4.0	***0.04***
Y1 Fibrinogen	−53.3 ± 14.3	***0.0002***	6.8 ± 3.9	0.08
Patients characteristics at baseline				
Age (per yr)	−13.1 ± 2.1	***< 0.0001***	0.9 ± 0.6	0.10
SEX (female vs male)	−358 ± 30.9	***< 0.0001***	6.8 ± 8.2	0.41
BMI (kg/m^2^)	10.7 ± 2.7	***< 0.0001***	0.2 ± 0.7	0.79
# prior AECOPD				
1 vs 0	−172 ± 35.0	***< 0.0001***	2.8 ± 9.6	0.77
2 vs 0	−151 ± 42.8	***0.0004***	18.9 ± 11.8	0.11
≥ 3 vs 0	−323 ± 42.2	***< 0.0001***	13.9 ± 13.8	0.31
AECOPD FU	Na	Na	−1.8 ± 1.9	0.35
Current smoker (yes vs no)	−19.4 ± 32.1	0.55	−30.7 ± 8.5	***0.0003***
Bronchodilator reversibility (yes vs. no)	241 ± 34.3	***< 0.0001***	−22.8 ± 9.1	***0.012***
Significant emphysema (yes vs. no)	−347 ± 31.6	***< 0.0001***	−27.9 ± 8.4	***0.001***
Chronic bronchitis (yes vs no)	−35.4 ± 31.1	0.26	−9.7 ± 8.2	0.24
#Pack years	−0.8 ± 0.6	0.17	0.1 ± 0.2	0.68

*CC-16* Club-cell secretory protein-16, *SP-D* Surfactant protein-D, *WBC* white blood cell count, *C1M* MMP derived collagen type I fragment, *C6M* MMP derived collagen type VI fragment, *hsCRP* high sensitive C-reactive protein, *BMI* Body mass index, *AECOPD* Acute exacerbations in COPD, *FU* Follow up, ^a^The association are calculated as per increase in 1SD in each biomarker or as indicated for each patient characteristics. na: non applicable

all *p* values below <0.05 should be bold and italic

### Association between clinical patient characteristics or single biomarkers and annual FEV_1_ change

**Fig. 4:**
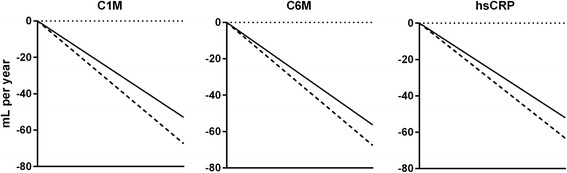
Estimate of annual loss in FEV_1_ having a serum biomarker concentration at the level of the 25th percentile (*dashed line*) and 75th percentile (*full line*). C1M: 25 percentile 46.3 ng/mL; 75 percentile: 105.5 ng/mL; C6M: 25 percentile 11.7 ng/mL; 75 percentile: 22.5 ng/mL, hsCRP: 25 percentile 1.50 ng/mL; 75 percentile: 7.45 ng/mL. Subject characteristics: Male, 63 years of age, BMI 26.9 kg/m2, former smoker with a history of 43 pack-years of smoking, 1 exacerbation in the previous 12 months, no exacerbations during follow-up, bronchodilator irreversible, and absence of emphysema and chronic bronchitis

The effect estimate of a 1SD change in a biomarkers was calculated for each marker in the total population for the PD-FEV_1_ and PD-FEV_1_% linear change from Y1 to Y3 (Table 2). An overview of the association for significant parameters is seen in Fig. 3, generally showing that patient characteristics and biomarkers were able to describe annual change in FEV_1_ with patient characteristics providing negative associations and serological markers providing positive associations. Serum C1M and C6M at 6 months were independent predictors of lung function change. A 1SD increase in C1M accounted for a decreased rate of decline of lung function of 10.4 mL/yr. (*p* = 0.007) in PD-FEV_1_. For C6M, a 1SD increase accounted for a a decreased rate of annual decline in lung function of 8.6 mL/yr. (*p* = 0.03) in PD-FEV_1_. The rate of change in PD-FEV_1_% for C1M was +0.37% (*p* = 0.005) and for C6M +0.29% (p = 0.03). The association between C1M and C6M and PD-FEV_1_ and PD-FEV_1_% change was not affected by baseline age, sex, GOLD stage, smoking status, emphysema, bronchodilator reversibility, and number of previous exacerbations. Y1 plasma hsCRP accounted for a rate of change in PD-FEV_1_ of 8.2 mL (*p* = 0.04) and 0.30% for PD-FEV_1_% (p = 0.03). Smoking status, bronchodilator reversibility, and emphysema were also confirmed as predictors. Fibrinogen and CC-16 were not able to predict rate of Y1 to Y3 annual decline for PD-FEV_1_ nor PD-FEV_1_% in this subpopulation of the ECLIPSE study. The estimated annual loss in FEV_1_ using the multimarker random-coefficient model for C1M, C6M and hsCRP is shown in Fig. 4. Here the 25th- and the 75th percentile groups are plotted showing the estimated loss in these two groups. For C1M, the annual loss of FEV_1_ was higher in patients below 46.3 ng/mL (25th percentile group) versus in patients below 105.5 ng/mL (75th percentile group). For C6M and hsCRP, the 25th percentile was 11.7- or 1.5 ng/mL, respectively and the 75th percentile 22.5- or 7.45 ng/mL, respectively. There was no significant associations to PD-FEV_1_ change within different GOLD stages, or in patients either being slow- or rapid decliners. A multimarker model including C1M, C6M, fibrinogen and hsCRP did not increase the association to linear change in PD-FEV_1_ compared to the use of single markers.

## Discussion

To the best of our knowledge, the present study is the first of its kind to evaluate the ability of novel serological ECM neoepitope markers, reflecting collagen type I and VI degradation, to predict the rate of change in lung function in patients with COPD. We demonstrated that serum C1M and C6M were independent predictors of lung function change in COPD patients over a two year period follow up. Furthermore, a significant correlation at baseline to FEV_1_ by several markers and patient characteristic was observed within the study.

The average annual rate of change in lung function was evaluated in the ECLIPSE study reporting a FEV_1_ decline of 33 ml per year, including patients that declined, were stable or improved in FEV_1_ [36]. Several clinical trials have shown that FEV_1_ decline was higher in mild COPD compared to more severe disease [40–43]. Such observations imply that COPD encloses a variety of patients with diverse FEV_1_ trajectories [44]. Thus, it has become a challenge to stratify patients into phenotypes such as slow and rapid FEV_1_ decliners_._ Better patient stratification and phenotyping by disease activity biomarkers would provide a highly valued tool for patient handling in general practice and in clinical trials to enrich the patient population [45].

Non-serological markers such as the "Body-mass index, airflow Obstruction, Dyspnoea, and Exercise" index (BODE), walking tests, exacerbation rate and dyspnoea score are known to be related to disease progression defined by loss of FEV_1_ and mortality in patients with COPD [36, 39]. However, there is a medical need for serological markers of progressive COPD and selection of patient phenotypes at baseline [46]. It was previously shown in the ECLIPSE (Evaluation of COPD Longitudinally to Identify Predictive Surrogate Endpoints) study [36] that patient characteristics, but not most serological biomarkers, were associated to change in lung function over the three-year period of the study. The analysis of biomarkers included baseline assessed fibrinogen, interleukin-6, interleukin-8, TNF-a, high sensitive C-reactive protein (hsCRP), Club Cell protein-16 (CC-16) and surfactant protein D in a subset of 1793 patients. Only CC-16 was significantly associated with lung function change. Increase of one standard deviation of CC-16 was equal to the protection of 4 ml/yr. (*p* = 0.04) in lung function change. hsCRP was only borderline significant (*p* = 0.07), but indicated that increased levels were associated with a lower rate of lung function decline.

Most serological markers evaluated in stable COPD, have shown limited value in relation to the pathology in COPD including sputum neutrophils, circulating white blood cells (WBC), and protein biomarkers [47]. Krebs von den Lungen-6 (KL-6), CC-16, surfactant protein-A (SP-A) and surfactant protein-D (SP-D) are discussed as serological markers of pulmonary diseases [48–51]. They have, however, not been convincingly demonstrated to be of value for patient phenotyping in COPD [48–51]. Desmosine and isodesmosine, two molecules involved in elastin cross-linking [48], have shown some value [52], but have not been fully validated for use in COPD. A marker of blood clotting, fibrinogen, was recently approved by the Food and Drug Administration (FDA) as a drug development tool for the use of patient enrichment in clinical intervention trials [53]. Fibrinogen appears to be related to COPD exacerbations and mortality and used as an aid in selection of patients that are most likely to progress to a clinical outcome that may be used as a hard endpoint [53].

At present, it has not yet been investigated whether pulmonary structural changes in the lung ECM in COPD is interrelated to rate of change in lung function. In a small number of patients with mild COPD, it has been shown that C1M and C6M were elevated when compared to healthy controls [54]. Furthermore, important data have been presented showing that these ECM neoepitope markers are related to exacerbations [32] and mortality in COPD [55]. A range of serological biomarkers have previously been evaluated in the full ECLIPSE study as predictors of annual rate of change in FEV_1_, yet only CC-16 was associated with a decreased rate of annual decline in lung function with a modest effect estimate of +4 ± 2 mL/yr. FEV_1_ per 1 SD change in plasma CC-16 [36, 56]. In the present study, serum C1M, C6M and plasma hsCRP were significantly related to a decreased rate of annual decline in lung function ranging from +8.2 to +10.4 mL/yr. FEV_1_ per 1SD change in each marker during a two year follow up, which was superior to the association observed for CC-16 [36]. In the subpopulation analysed in the present work, CC-16 was not significantly related to the rate of lung function change. It was notable that increasing levels of all the significant related markers including CC-16 in the full ECLIPSE study [36] were related to a lower rate of lung function decline which may indicate that patients with impaired tissue repair, thus low biomarker levels, progress the fastest in relation to lung function decline. This is however speculative at present. The data did not change when patients were stratified according to GOLD stage C-D or being slow- or rapid lung function decliners. These data were nevertheless unexpected since we intuitively regard elevated levels of ECM remodelling and CRP as being related to poor outcome in patients. In the literature it has been presented that serum hsCRP was prognostic of mortality in stable COPD [57, 58] and that persistent systemic inflammation, here among others plasma CRP, is associated with poor clinical outcome in the ECLIPSE cohort [59]. Nevertheless, persistent inflammation was not related to the prevalence of chronic bronchitis, degree of emphysema, bronchodilator reponse and rate of FEV_1_ decline in the population investigated [59]. Finally, WBC, C1M, C6M, hsCRP and Fibrinogen were associated with a lower baseline FEV_1_ indicating that severe patients according to lung function have higher biomarker levels, correlating to the data published on death outcome [55], as previously mentioned. Lange et al. [60] has published how the effect of small lungs from early age has an effect on FEV_1_, thus an effect on diagnosis of COPD, and potentially COPD phenotypes, and FEV_1_ change. The ability of predicting annual rate of change in FEV1 may be different in mild COPD vs moderate to severe but also depend on baseline FEV_1_.

In a small study of COPD patients, Ferrari et al. indicated that plasma hsCRP was not related to mortality [61], thus data are to some extent contradictory. Inconsistently to the data in the present publication, Shabann et al. revealed that the highest tertile of serum CRP levels was associated with greater decline in annual FEV_1_ in COPD patients [62], which was not the conclusion of the present work. The discrepancy between the two studies may relate to the large differences in age, BMI and FEV_1_ at study entry. The COPD patients investigated by Shaaban et al. [62] were younger (mean 36.6 yr), more lean (BMI = 22.6) and had a higher FEV_1_% (105%), however had similar rate of annual decline in lung function (33 vs 29 ml). Our findings indicate that various patohology linked pathways in COPD have a distinct effect on the ECM remodelling thus levels of ECM neo-epitopes. Finally, in the ECLIPSE study analysis, we have observed that C1M, C6M and hsCRP are elevated in patients that died within the study period compared to those who did not {Sand, 2016 4514 /id, which indicates that high levels of such markers are related to a poor outcome.

In the present study, there was no relation between the structure related markers C1M and C6M or CRP with having significant emphysema or not. This may be explained by the fact that the emphysema defined groups are still very heterogeneous and have different ECM remodelling profiles. It appears that assessment of MMP degraded collagen type I or type VI and total CRP are not the correct markers to evaluate emphysema in a broad population.

The stability of C1M and C6M has in a large cohort of patients with IPF been shown over a six month time period [21]. Here it was seen that levels were stable over time and that patients within the high level or low level group at baseline stayed within this group over time. Nevertheless, it was seen that a relative small increase of C1M and C6M over time in individual IPF patients were related to loss of FVC or death {Jenkins, 2015 4101 /id}. These data, eventhough being in IPF, which is a fibrogenesis related disease, are contradictory to the present findings in COPD.

We confirmed in this subpopulation that smoking status, bronchodilator reversibility, and emphysema were significantly related to the annual rate of FEV_1_-decline ranging between −30.7-27.9 mL/yr. A pronounced and significant association by both serological markers and patient characteristics was seen on baseline FEV_1_. Serum C1M and C6M, plasma CC-16, hsCRP, and fibrinogen, age, sex, BMI, prior exacerbations, bronchodilator reversibility and emphysema had a significant association with baseline lung capacity. This highlights the importance of these markers and characteristics in the description of COPD traits and potentially their role in patient stratification. The use of a single marker reflecting few pathophysiological traits may not be sufficient whereas a panel of markers most likely may be required for optimal prognosis assessment and evaluation of efficacy of intervention of upcoming drugs. However, neither C1M nor C6M did in the multimarker model increase the association with annual change.

### Limitations

In general, the rate of decline in FEV_1_ observed within the ECLIPSE study was small and highly variable [36] providing a small window of progression during the two year period. Furthermore, FEV_1_ measurements are generally recognized as being highly variable. In the present study, the two extreme groups in FEV_1_ change were selected for analysis thus leaving out the patients with FEV_1_ changes in-between; this may have added to lowering data noise observed in real world COPD studies. Generally, the analysis of serological biochemical markers using the ELISA technique often has limitations such as background noise from the normal level of tissue remodelling and variation inherited into the method. Here the signal-to-noise ratio may be improved by incorporation of disease-related post-translational modifications such as protease cleavage into the specificity criteria of a given assay [63].

In perspective, novel protein fingerprint fibrosis markers as evaluated in the present study may provide a new clinical opportunity for improved evaluation of the involvement of small airways fibrosis activity in COPD. This may add to phenotype stratification and selection of patients for clinical trials. However, this needs to be proven in future prospective studies. Nevertheless, the data at present are preliminary and verification in a second cohort is needed including evaluation in milder COPD. Furthermore, data should additionally be evaluated in a “real world” COPD study where the criteria of extreme annual rate of FEV_1_ is not included.

## Conclusion

In conclusion, we demonstrated that extracellular matrix markers of tissue turnover were predictive of FEV_1_ change in patients with COPD and suggest that such markers may be used as prognostic biomarkers and possibly biomarkers for patient enrichment in clinical trials.

## Additional files

Additional file 1: Details of IRB approval sites. No data, this is a list of the local ethical committees that approved the study. (DOC 80 kb)

Additional file 2: Members of the ECLIPSE steering committee. No data, this is text describing the ECLIPSE members and investigators. (DOCX 14 kb)

## Abbreviations

%LAA: Percentage of lung CT voxels below a threshold of −950 Hounsfield units and percent low areas of attenuation
1SD: One standard deviation
ATS-DLD: American thoracic society–division of lung disease
BL: Baseline
BODE: Body-mass index, airflow obstruction, dyspnoea, and exercise index
C1M: Marker of MMP degraded type I collagen
C6M: Marker of MMP degraded type VI collagen
CC-16: Club Cell protein-16
COPD: Chronic obstructive pulmonary disease (COPD)
CT: Computed tomographic
ECLIPSE: Evaluation of COPD longitudinally to identify predictive surrogate endpoints study ELISA Enzyme linked immunosorbent assay
ECM: Extracellular matrix
FDA: Food and drug administration
FEV_1_: Forced expiratory volumen in 1 s
FVC: Forced vital capacity)
GOLD: The global initiative for obstructive lung disease
hsCRP: High sensitive C-reactive protein
IPF: Idiopathic pulmonary fibrosis
KL-6: Krebs von den Lungen-6
M6: Month 6
MMP: Matrix metalloproteinase
PD-FEV_1_: Post-dose bronchodilator FEV_1_
SP-A: Surfactant protein-A
SP-D: Surfactant protein-D
WCB: White blood cells
